# Evaluating Angiogenic Potential of Small Molecules Using Genetic Network Approaches

**DOI:** 10.1007/s40883-018-0077-8

**Published:** 2018-09-27

**Authors:** Anusuya Das, Parker Merrill, Jennifer Wilson, Thomas Turner, Mikell Paige, Scott Capitosti, Milton Brown, Brandon Freshcorn, Mary Caitlin P. Sok, Hannah Song, Edward A. Botchwey

**Affiliations:** 1 Department of Orthopaedic Surgery, University of Virginia, Charlottesville, VA, USA; 2 Department of Biomedical Engineering, University of Virginia, Charlottesville, VA, USA; 3 Department of Biological Engineering, Massachusetts Institute of Technology, Cambridge, MA, USA; 4 Department of Biomedical Engineering, Georgia Institute of Technology, Atlanta, GA, USA; 5 Parker H. Petit Institute for Bioengineering and Bioscience, 315 Ferst Drive Suite 1316, Atlanta, GA 30332, USA; 6 Center for Drug Discovery, Georgetown University, Washington, DC, USA; 7 School of Medicine, University of Virginia, Charlottesville, VA, USA

**Keywords:** PNF-1, Pathway compendium analysis, Phthalimide compounds

## Abstract

Control of microvascular network growth is critical to treatment of ischemic tissue diseases and enhancing regenerative capacity of tissue engineering implants. Conventional therapeutic strategies for inducing angiogenesis aim to deliver one or more proangiogenic cytokines or to over-express known pro-angiogenic genes, but seldom address potential compensatory or cooperative effects between signals and the overarching signaling pathways that determine successful outcomes. An emerging grand challenge is harnessing the expanding knowledge base of angiogenic signaling pathways toward development of successful new therapies. We previously performed drug optimization studies by various substitutions of a 2-(2,6-dioxo-3-piperidyl)isoindole-1,3-dione scaffold to discover novel bioactive small molecules capable of inducing growth of microvascular networks, the most potent of which we termed phthalimide neovascularization factor 1 (PNF1, formerly known as SC-3–149). We then showed that PNF-1 regulates the transcription of signaling molecules that are associated with vascular initiation and maturation in a time-dependent manner through a novel pathway compendium analysis in which transcriptional regulatory networks of PNF-1-stimulated microvascular endothelial cells are overlaid with literature-derived angiogenic pathways. In this study, we generated three analogues (SC-3–143, SC-3–263, SC-3–13) through systematic transformations to PNF1 to evaluate the effects of electronic, steric, chiral, and hydrogen bonding changes on angiogenic signaling. We then expanded our compendium analysis toward these new compounds. Variables obtained from the compendium analysis were then used to construct a PLSR model to predict endothelial cell proliferation. Our combined approach suggests mechanisms of action involving suppression of VEGF pathways through TGF-β andNR3C1 network activation.

## Lay Summary

Previously, we discovered a novel small molecule (PNF1) that is capable of inducing growth of microvascular networks, a mechanism that is very important in many regenerative applications. In this study, we alter the structure of PNF1 slightly to get three different analogues and focus on gaining insight into how these drugs induce their pro-angiogenic effects. This is done through a few techniques that result in a map of all the transcripts that are up- or downregulated as a result of administering the drug, a knowledge that is necessary for successful therapeutic strategies.

## Future Studies

Angiogenesis and neovascularization is important in a number of regenerative medicine therapeutics, including soft tissue regeneration. Having a deep understanding of the transcriptional mechanism of small molecules with this angiogenic potential will aid in designing specific immunomodulatory biomaterials. In the future, we will study these drugs and their angiogenic properties in impactful and clinically translatable applications.

## Introduction

Despite the recent advancements in drug-induced neovascularization, further development of drug therapies is needed for improvement of functional vessel systems [1]. The creation of functional microvascular networks requires many endogenous growth factors to initiate vessel sprouting and remodeling and to regulate cellular interactions [2]. Consequently, a number of growth factors have been studied to increase angiogenesis during wound repair. In particular, initial efforts for drug-induced revascularization have been focused on peptide-based growth factors such as recombinant vascular endothelial growth factor (VEGF). These biological molecules hold significant promise for developing effective therapies for neovascularization; however, these treatments have many significant drawbacks, including the high cost of protein recovery and purification, as well as susceptibility to aggregation and degradation in the human body [3, 4]. Moreover, large doses of these growth factors carry risks of hyperstimulation and angioma formation [5]. For this reason, significant interest is emerging in the use of synthetic small molecules for the induction of angiogenesis [6].

In previous studies, we discovered a novel small molecule, phthalimide neovascularization factor 1 (PNF-1 or SC-3–149), which is a potent stimulator of microvascular network growth [7]. PNF-1 possesses a molecular weight similar to that of known small molecule therapeutics. It may be well suited for localized drug delivery applications given its small size compared to that of peptide-based growth factors, including VEGF (229 amu and 38.2 kDa, respectively). In addition, while processing methods used for the incorporation of molecules into polymeric biomaterials may compromise peptide-based growth factors, this small molecule is better suited to withstand them [8].

We then showed that PNF-1 regulates the transcription of signaling molecules that are associated with vascular initiation and maturation in a time-dependent manner through a novel pathway compendium analysis. In this analysis, transcriptional regulatory networks of PNF-1-stimulated microvascular endothelial cells are overlaid with literature-derived angiogenic pathways [7, 9−11]. Parallel in vivo experimental quantification of systems-level remodeling of microvascular network growth induced by PNF-1 delivery revealed a similar sequential pattern of inflammation-mediated microvascular initiation, expansion, and subsequent maturation. Intravital imaging analysis of intact microvessel networks show that PNF-1 induces increases in total microvessel length density at early time points, followed by changes in the luminal diameter of local arterioles and other hallmarks of vascular maturation. [7,9].

In this study, we validated our pathway compendium analysis to generate new transcriptional regulatory network m odels from hum an m icrovascular endothelial cells (HMVECs) over multiple time points after PNF-1 stimulation. Network analysis by Ingenuity Pathway Analysis (IPA, Redwood City, CA, USA) was used to examine possible differences between our original analysis results owing to the expansion of the Ingenuity Pathway Knowledge Base (IPKB). Moreover, we evaluated the effects of other structurally similar investigational compounds analogous to PNF-1 that show mitogenic effects in human microvascular endothelial cells. We modeled these effects, namely cell proliferation, using a partial least squares regression (PLSR) model with data obtained from the compendium analysis. Even with the large amount of highly colinear parameters, PLSR allows us to construct an accurate predictive model. By comparing the relative “fingerprints” of each angiogenic pathway of four structurally similar small molecules, we elucidate shared mechanisms that allow for functional molecular grouping or phenotyping.

This approach can increase the understanding of the complex balance between these protagonistic and antagonistic cues resulting in microvascular network growth. Identifying the balance between pro- and anti-angiogenic factors, the so called “angiogenic switch” may serve to enhance efforts at directed drug discovery of pro-angiogenic therapeutics.

## Materials and Methods

### Small Molecule Drugs

**Fig.1:**
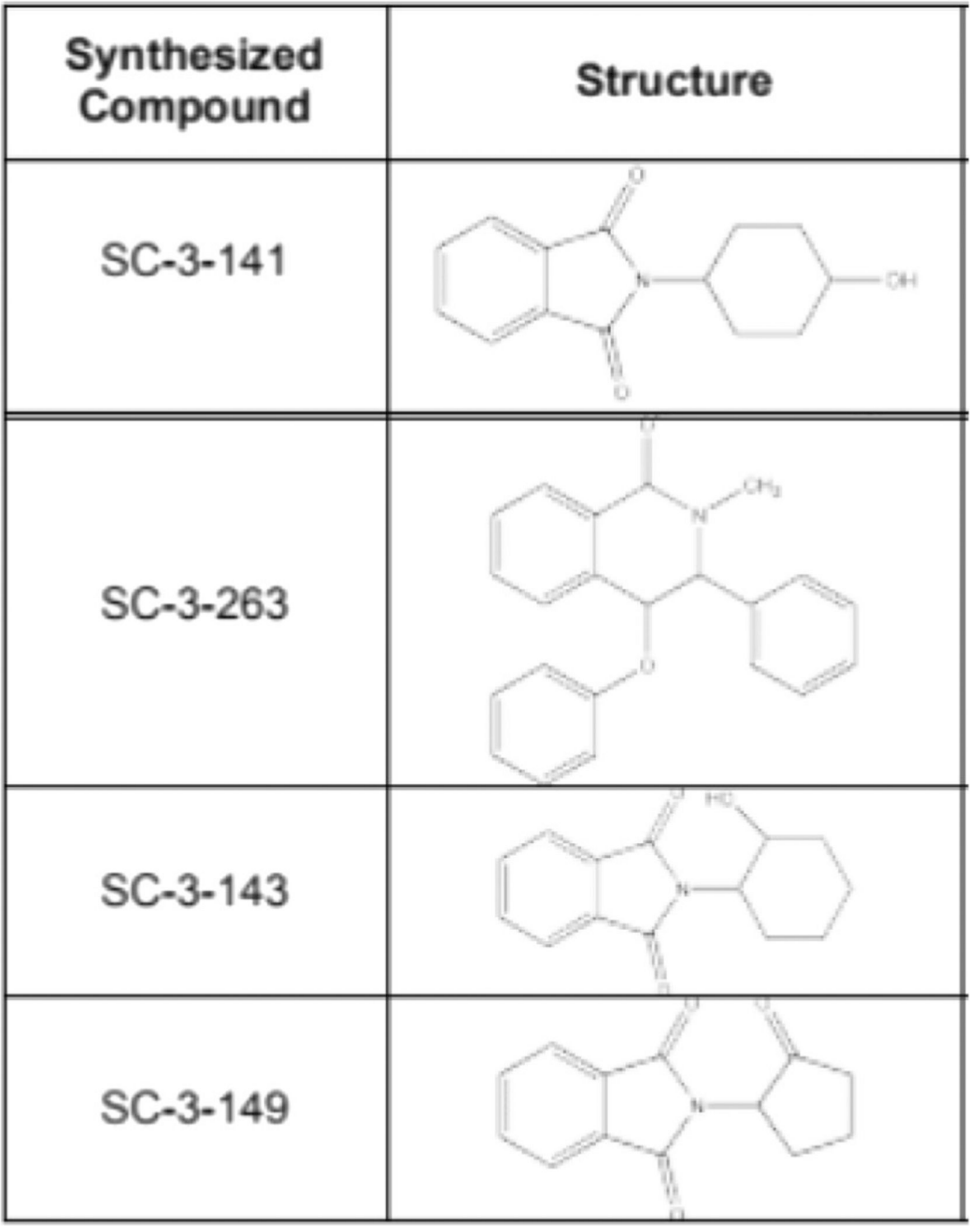
Small molecule structures. Novel bioactive molecules capable of inducing growth of microvascular networks

Four pthalidomide-based small molecules drugs were used in this study. Previously, we performed drug optimization studies by various substitutions of a 2-(2,6-dioxo-3-piperidyl)isoindole-1,3-dione scaffold to discover novel bioactive small molecules capable of inducing growth of microvascular networks, one of which we termed PNF-1. For this study, we generated new investigational compounds through systemic transformations to PNF-1 to evaluate the effects of electronic, steric, chiral, and hydrogen bonding changes on the endothelial cell phenotype behavior elicited by the drugs (Fig. 1). Though PNF-1 has been extensively studied as a pro-angiogenic agent both in vitro and in vivo, the other three drugs, SC-4–141, SC-3–143, and SC-3–263, are relatively unstudied molecules that also show promise in the field of neovascularization [7, 9, 12].

### Cell Culture

Human microvascular endothelial cells (HMVECs) (Lonza-Walkersville) were cultured in endothelial growth medium 2-microvascular (EGM-2-MV) (bulletkit, Lonza-Walkersville) supplemented with 5% fetal bovine serum. Cells were cultured on p100 tissue culture plates at 37 °C in a humidified chamber with 5% carbon dioxide.

### Proliferation Assay and RNA Isolation

HMVECs were plated at 2.5 × 10^4^ cells/cm^2^ on p100 tissue culture plates, grown to confluence and subjected to 30 μM of the treatment group’s respective drug, which affects the proliferation of HMVECs due to their angiogenic properties [10]. The 30-μM treatment concentrations were previously determined to influence the greatest increase in HMVEC proliferation [13]. Treatment groups were PNF-1, SC-3-141, SC-3-143, and SC-3-263, with VEGF and endostatin as the positive and negative controls, respectively. Total cells were counted via flow cytometry using the Guava ViaCount Assay (www.millipore.com) immediately prior to treatment and at the 24-h endpoint. Twenty-four hours after stimulation, total RNA was isolated using an RNeasy isolation kit (Qiagen, Inc.) according to the manufacture’s protocol.

### Gene Microarray Processing and Analysis

RNA samples were prepared for hybridization using the GeneChip One-Cycle Labeling and Control Reagents kit (Affymetrix). Expression profiles were created using GeneChip Human Genome U133 Plus 2.0 Array (Affymetrix), which contains a total of 47,000 human gene transcripts and variants. Expression profiles for PNF-1 treated HMVECs at seven time points (1, 2, 4, 8, 16, 24, and 48 h) were retrieved from the Gene Expression Omnibus [9]. Data pre-processing was performed using MATLAB scripts for background subtraction, normalization, and robust multi­array analysis (RMA) summarization (Mathworks, Natick, MA, USA). *p* values for differential expression were calculated based on the Wilcoxon signed-rank test, and significant differentially expressed genes were selected with *p* values below 0.003. Replicate gene IDs were then removed and their collective *p* values averaged. Microarray processing resulted in between 600 and 1400 differentially expressed genes per drug treatment group. Differentially expressed genes for the seven PNF-1 time points were compared in MATLAB using the pathway compendium analysis presented previously [11]. This analysis was repeated for SC-3–141, SC-3–143, SC-3­263, VEGF, and endostatin after 24 h of treatment.

### Gene Ontology Network Analysis

IPA was used in conjunction with the IPKB for gene network analysis. IPA has been used in previous gene network studies on microvascular remodeling as well as cellular responses to small molecules [3, 7, 14, 15]. The identified lists of significantly differentially regulated genes for each treatment group were uploaded into IPA and filtered based on gene eligibility for functional analysis. These remaining genes, called “focus genes,” were then used in all following IPA functions.

#### Identification of Common Nodes

Common nodes between treatment groups were identified using differential expression data. Common node comparisons are the simplest method for expression comparisons and do not require any statistical testing beyond differential expression. A gene is considered a common node if it is shared as a focus gene in two or more drug profiles.

#### Identification of Upstream Regulators

Predicted upstream regulators of downstream focus genes were identified for each treatment group using the “upstream regulators” function. IPA identifies the upstream transcription factors that can explain the differential gene expression shown in experimental data. Confidence in activation or inactivation of upstream regulators is expressed via *p* values using Fisher’s exact test, which calculates the significance of enrichment of the gene expression data for genes downstream of an upstream regulator. The upstream regulators method was determined to be a more comprehensive method of identifying mechanistic overlap because of its inclusion of literature-derived genetic relationships in its scoring algorithms.

#### Identification of Top Genetic Networks and Functional Analysis

Top genetic networks for each treatment group were constructed based on literature-based node connections. Networks were generated and scored based on their connectivity of focus genes. Networks were ranked based on their IPA given scores, which represent the probability that each isolated network of genes could be achieved by chance alone. Scores greater than three have a 99.9% confidence level of not being generated by random chance. The top three networks for each treatment group were selected for further analysis outside of IPA. These top three networks were then analyzed using the “canonical pathways” and “functional analysis” tools.

### Compendium Analysis

To further analyze the genetic profiles of the drugs with a specific emphasis on angiogenesis, we applied the network compendium presented by Wieghaus et al. to all four datasets [11]. Using Ingenuity, molecular interactions specific to each selected pathway—angiopoietin 1 (Ang1), chemokine ligand 2 (CCL2), basic fibroblast growth factor (bFGF), platelet-derived growth factor (PDGF), placental growth factor (PGF), TGF-β, tumor necrosis factor-alpha (TNF-α), vascular endothelial growth factor (VEGF), and glucocorticoid receptor (NR3C1)—were quantified and assigned an activation state of up- or downregulation.

Massive expansion of gene relationships can increase the total pool of interactions for each compendium category, thereby reducing the percentage calculations across the board. A percentage normalization method was used to provide an easier way to view the up-to-downstream regulation ratio, which is difficult to compare via the total genes method.

### Partial Least Squares Regression

For the construction of the PLSR model, 18 predictor variables (*X*) were used, corresponding to the up- and downstream activation of each of the nine pathways specified in our compendium analysis. The cell growth rate data collected for each drug were used as the observed response variable (*Y*). Gene expression data and cell proliferation data from five treatments (SC-3–141, SC-3–263, SC-3–143, VEGF, and endostatin) were used to create the model. The model was created using MATLAB’s *plsregress* function on a 5 by 18 matrix of gene expression data and a 5 by 1 array of cell growth values. The resulting model was then applied to the gene expression data of each of the six treatments to predict its cell growth output, including PNF-1, which was not used in the model creation. The model underwent sensitivity testing to identify variables with an aberrant effect on prediction. A total error score was calculated using the residual sum of squares. The resulting model was reapplied after the removal of predictor variables that reduced the percent error.

## Results

### Novel Drugs Exhibit Elevated Proliferation Rates

**Fig. 2:**
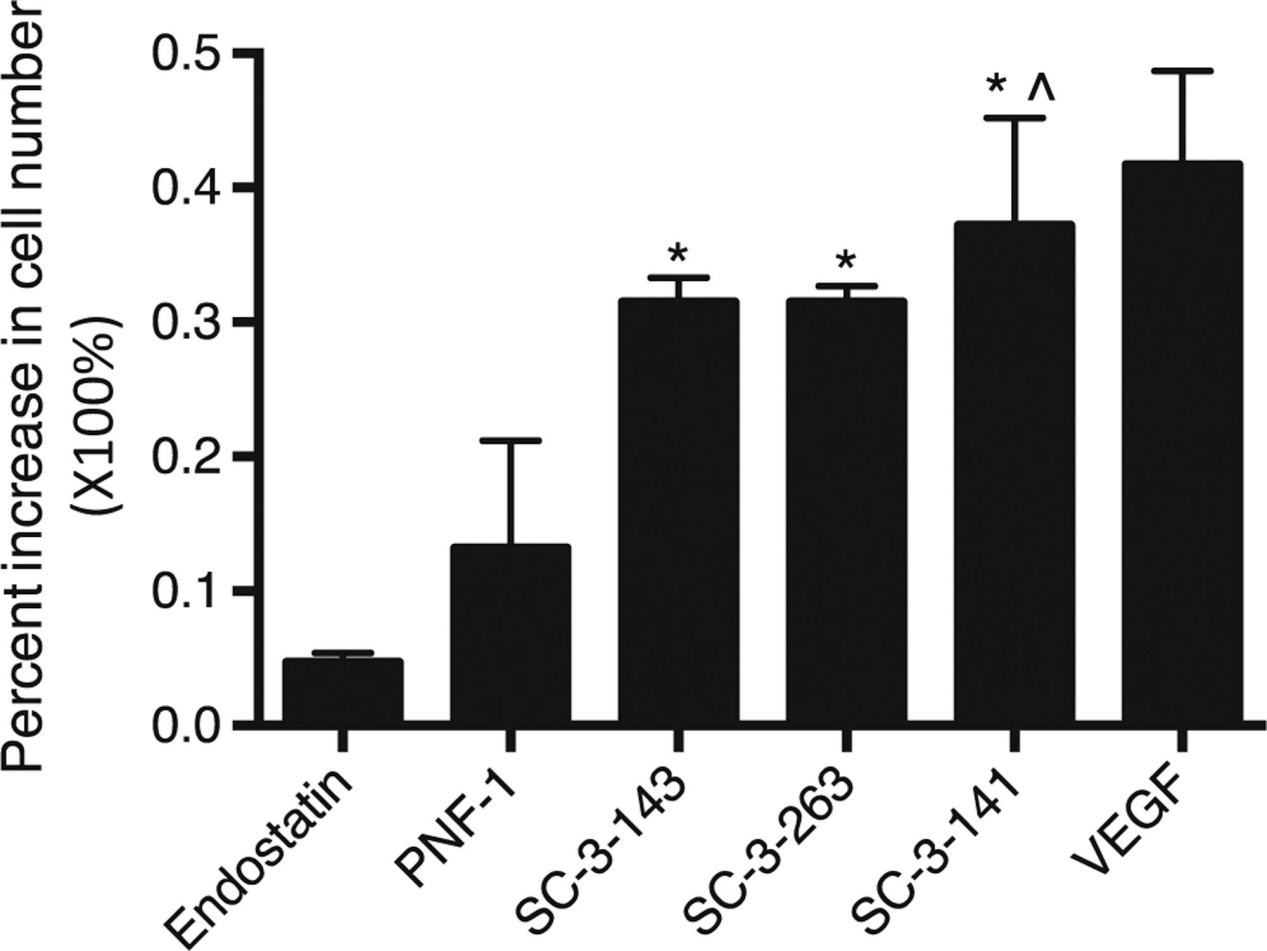
Impact on HMVEC proliferation. Percent increase of cell number compared to no-treatment control group after 24 h of exposure to 200 ng/mL of treatment drugs. VEGF and endostatin were used as positive and negative controls, respectively (*p < 0.05)

In vitro proliferation assays were conducted to assess the phenotypic responses induced by each drug. Figure 2 shows the effect of different treatment on HMVEC proliferation as measured by Guava ViaCount assay. Significance was determined using Student’s *t* test with a *p* = 0.05. All treatment groups show significantly increased doubling rates when compared to the vehicle control except for endostatin. In addition, all treatment groups significantly increased doubling rates over the endostatin negative control. Among novel treatments, only SC-3–141 exhibited significant doubling rates over PNF-1. VEGF treatment resulted in the highest cell count.

### Novel Drugs Exhibit Limited Node Commonality

Microarray data pre-processing resulted in between 600 and 1400 differentially expressed genes for each treatment group. The total numbers were reduced further with IPA’s selection of focus genes. IPA’s database of genes available for functional analysis has improved drastically in recent years. Previously, gene eligibility for PNF-1 was 47%, compared to 86% shown in this study (Table 1) [9]. This considerable increase in database size could result in significant changes in the network analysis.

Following focus gene selection, datasets were compared to find overlapping differentially expressed genes. Table 2 shows the absolute numbers for total genes shared between treatment groups. This common node selection did not yield any significant differences in dataset comparison between sets of two treatment groups, although SC-3–143 and SC-3–263 showed the largest gene overlap at 9.3%. Percentages remained low (< 10%), and no individual nodes were shared between all groups. This analysis suggests limited overlap of specific gene stimulation between all drug treatments but does not rule out network or regulatory overlap.

### Upstream Regulation Shows Drug Similarity and Growth Factor Activation

Predicted upstream regulator activation for each treatment group was checked for overlap between groups to identify drugs with similar mechanisms. Tables 3 and 4 show total gene overlap for activated and inactivated upstream regulators, respectively. Overlap activation numbers suggest two of the drugs, SC-3–143 and SC-3–263, have a similar mechanism of action. This is consistent with the findings from the proliferation assay, which found that they induced similar doubling rates. Each treatment group was then analyzed individually to identify the most statistically significant upstream regulators. Statistical analysis in Ingenuity of gene activation resulted in *p* values smaller than 10^−10^ for all genes discussed below.

SC-3–263 exhibited several significant growth factor-related upstream regulators. Tumor necrosis factor-alpha (TNF-α) was one of the highest scoring molecules predicted to be activated, along with growth factors platelet-derived growth factor BB (PDGF BB, a dimer of PDGF), epidermal growth factor (EGF), and leukotriene, a regulator of inflammatory response. SC-3–143 had similar trends in upstream regulator activation, with predicted activation of TNF-α, PDGF BB, and β-estradiol, predominantly a sex hormone. β-estradiol plays many reproductive rolls, but it has also been shown to improve arterial blood flow in coronary arteries. SC-3–141 exhibited a mix of anti­cancer gene activation. The genes for TP53, a tumor suppressor protein, CDKN1A, which creates p21 protein and is tightly restricted by TP53, and 1-alpha, 25-dihydroxy vitamin D_3_ (calcitriol), were predicted as activated.

Many of the results of the upstream regulator analysis are consistent with pthalidomide-based small molecules. Because these drugs were originally synthesized with the intention of inhibiting cancer, it was not surprising that cancer-inhibiting upstream regulators such as TNF-α and TP53 were predicted to be activated. TNF-α has also been linked to new blood vessel formation suggesting a possible pro-angiogenic synergy with PDGF BB in SC-3–143 and SC-3–263.

### Network and Functional Analysis Elucidates Common Pathways

Individual nodes in each network were ranked for their total number of connections or molecular interactions within the network (Table 5). Concurrently, a functional analysis was performed in IPA on the merged networks and predicted canonical pathways were identified. The combined results are described below.

Cells treated with PNF-1 upregulated the cDNA of TGB-1, which promotes cellular proliferation and differentiation as previously reported, and P13k, a family of enzymes involved in responses to growth factors, proliferation, and differentiation [11]. In functional analysis, two canonical pathways were significant: glucocorticoid receptor signaling and TGF-β signaling. In these pathways, ten and six molecules were predicted to be activated in the network, respectively. Predicted functions for the network were cell cycle, cellular movement, cellular elongation, cellular cytokinesis, and cell morphology.

In the network analysis of SC-3–141-treated cells, the most central network was built around vascular endothelial growth factor (VEGF), most of which was downregulated (including VEGF). Conversely, NR3C1 (glucocorticoid receptor), a pleiotropic gene complex was found to be significantly upregulated. In functional analysis, the four most significant canonical pathways were glucocorticoid receptor signaling, molecular mechanisms of cancer, pancreatic adenocarcinoma signaling, and the role of endothelial cells in rheumatoid arthritis. These pathways each had nine to 11 activated molecules in the combined network. Predicted functions for this network were cell cycle, cellular assembly and organization, cancer, and proliferation.

In the network analysis of SC-3–143-treated specimens, immunoglobulin G (IgG), an antibody isotype, bone morphogenic protein 4 (BMP-4), a part of the TGF-β super­family, and P13k were predicted to be upregulated in their respective network. Subsequently, NF-kB, which controls DNA transcription in response to stress, free radicals, antigens, or cytokines, and ERK 1/2 both displayed a high number of interactions, but were not differentially expressed. In functional analysis, the four most significant canonical pathways were systemic lupus erythematosus, molecular mechanisms of cancer, glucocorticoid receptor signaling, and TGF-β signaling. As with PNF-1, these pathways were not largely activated, with only five to ten molecules activated in the combined network. Predicted functions for this network were RNA post-translational modification and vitamin D-resistant rickets.

Finally, in the network analysis of SC-3–263 treated specimens, cellular proto-oncogene (c-Fos), a transcription factor upregulated in response to many growth factors, and transforming growth factor-beta 1 (TGF-β1), which promotes cellular proliferation and differentiation, show a large number of connections and were concurrently upregulated. The SOS gene also showed a large number of molecular interactions but was not differentially expressed. In functional analysis, four canonical pathways stood out: the role of endothelial cells in rheumatoid arthritis, dendritic cell maturation, glucocorticoid receptor signaling, and TGF-β signaling. These pathways contained between ten and 20 activated molecules in the combined network. Top predicted functions for this network were morphology of cells, quantity of cells, and cellular differentiation. *p* values for confidence in network function all fell below 10^−10^.

**Fig.3:**
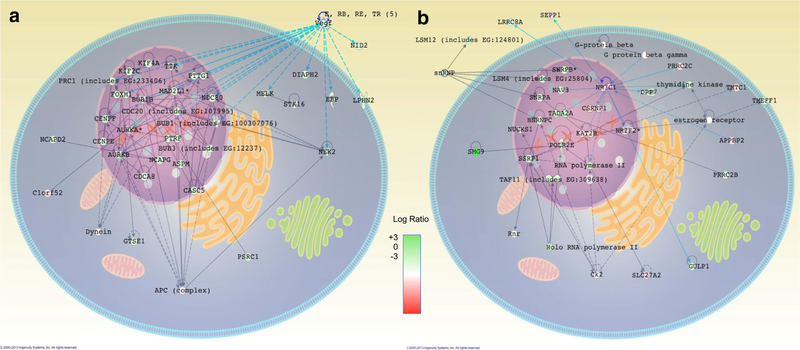
Identification of unique pathways after treatment with SC-3–141. Of all of pthalidomide-based molecules tested, SC-3–141 has the highest impact on HUVEC proliferation. **a** Downregulation of VEGF pathways is characteristic of pthalidomide-based molecules. All but one of the molecules in this network shows downregulation, demonstrating the effects of the drug are completely independent of this pathway. **b** Upregulation of the pleiotropic gene NR3C1 could be responsible for proliferative behavior, especially when linked to PDGF. Downregulation of SMG9 and a mixture of down- and upregulation in this network present a problem determining the exact mechanisms of NR3C1 contributions

Alongside PNF-1 networks, SC-3–143 and SC-3–263 have TGF-β as a common theme in the functional analysis of canonical pathways and show a significant TGF-β-centered mechanistic dependence. These relationships suggest that these pthalidom ide-based molecules may act through similar TGF-β-dependent receptors or pathways. Significant VEGF pathway downregulation present in SC-3–141 is also consistent with pthalidomide-based molecules; however, this is likely not responsible for any proangiogenic effects (Fig. 3a). SC-3–141 had significant up-regulation of the NR3C1 pathway (Fig. 3b), which appears in the functional analysis of canonical pathways for all four compounds. Pleiotropic genes such as NR3C1 show some of the greatest pro-angiogenic promise due to their combination of known and unknown pathways [6].

### Combined Network Demonstrates TGF-β Homogeneity

**Fig. 4:**
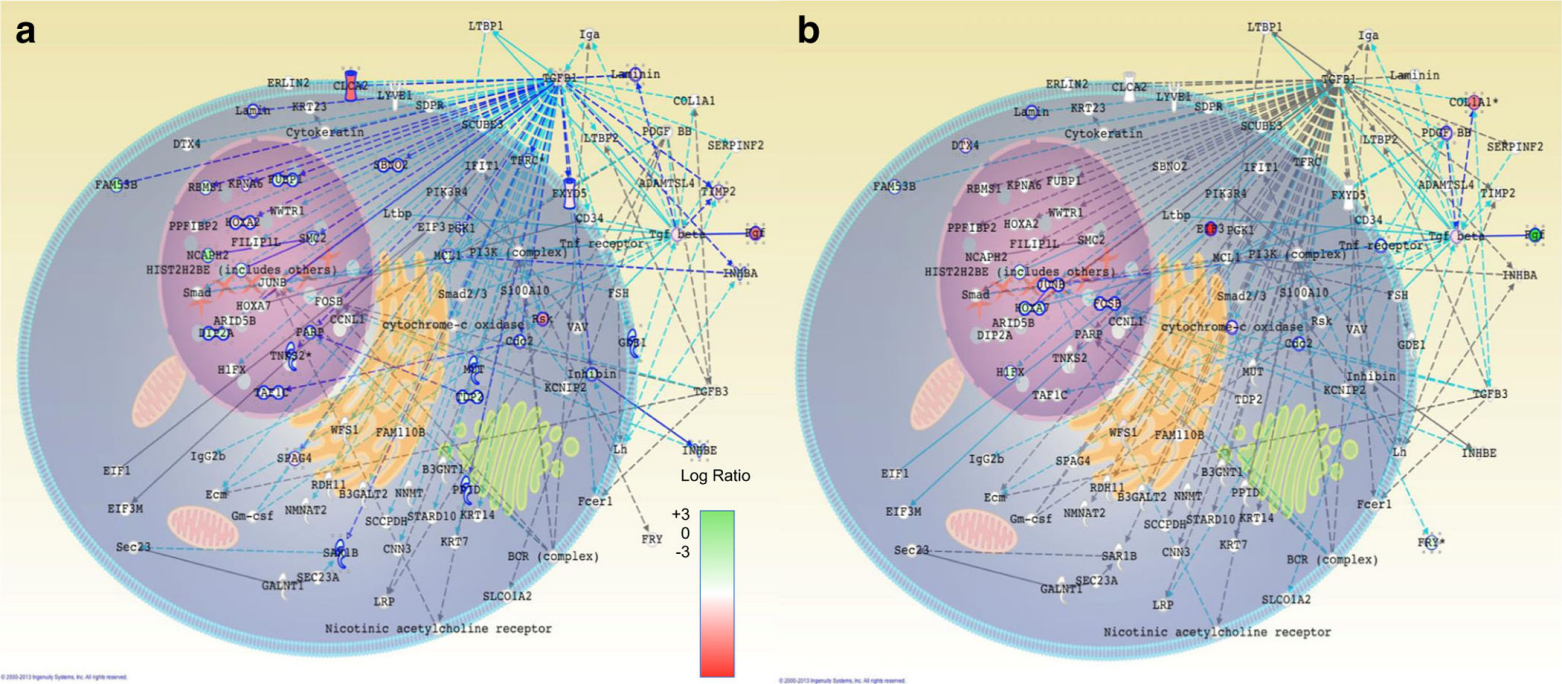
Merged TGF-β network after treatment with PNF-1 or SC-3–141 demonstrating the similarities and differences in the TGF-β pathways. **a** PNF-1 treatment results upregulation of all three TGF-β elements, as well as numerous downstream targets. In addition, FGF and CLCA2 are highly upregulated. **b** SC-3–141 treatment results in activation of just the TGF-β family, as well as COL1A1, EIF3, and PDGF BB. SC-3­141 also heavily downregulated FGF. Dark blue lines represent molecular interactions between two differentially expressed molecules, and light blue lines represent interactions between one differentially expressed and one non-expressed molecule. The spatial distribution of each gene represents the location of its corresponding molecule throughout the cell, and the coloration of the molecules represents the log ratio of the gene fold changes. Green indicates reduced expression and red indicates increased expression

**Fig. 5:**
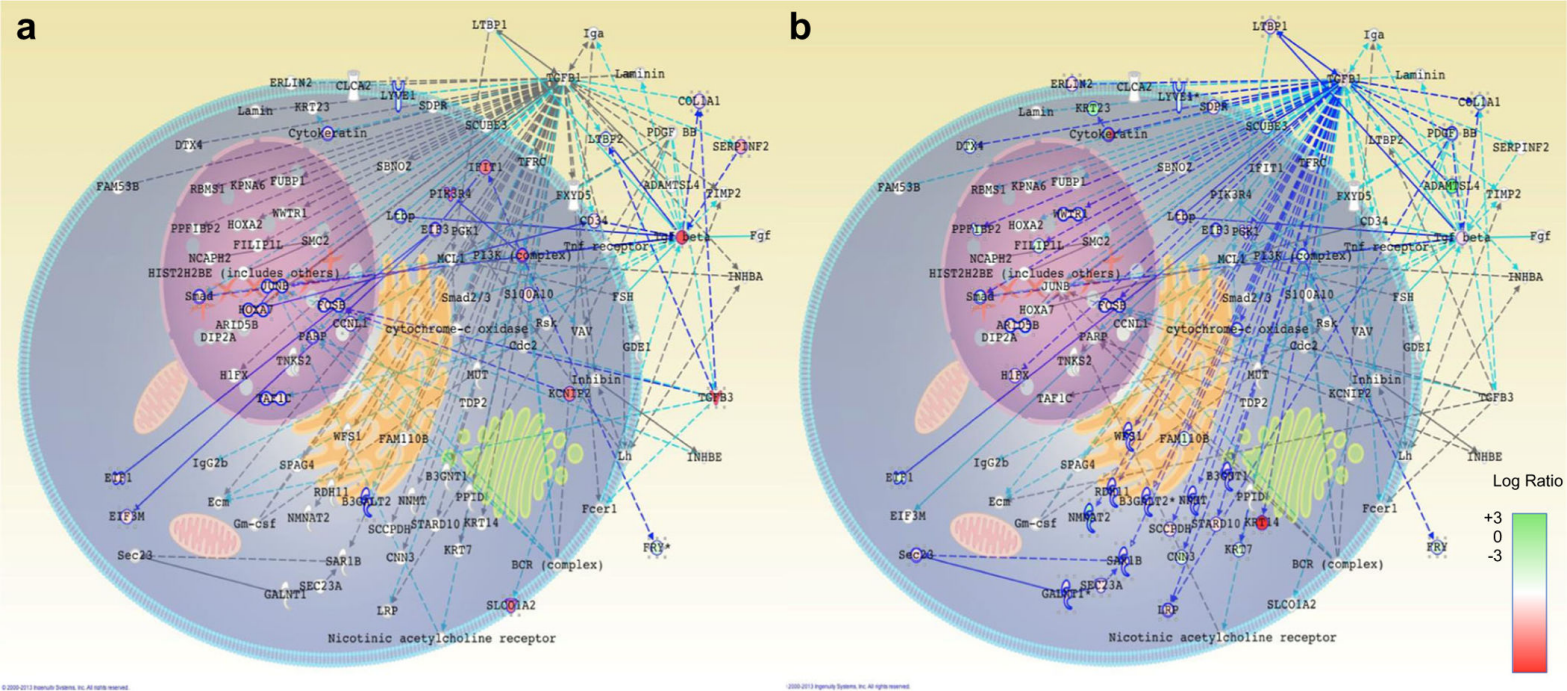
Merged TGF-β network after treatment with SC-3–143 or SC-3­263 demonstrating the similarities and differences in the TGF-β pathways: **a** SC-3–143 treatment results in heavy upregulation of the TGF-β family as TGFB3 elements. P13K, IFIT1, SERPINF2, and KCNIP2 are also upregulated. **b** SC-3–263 treatment results in TGFB1 and TGF-β family upregulation, as well as KRT14. SC-3–263 also downregulated PDGF BB and upregulated FGF. Dark blue lines represent molecular interactions between two differentially expressed molecules, and light blue lines represent interactions between one differentially expressed and one non-expressed molecule. The spatial distribution of each gene represents the location of its corresponding molecule throughout the cell, and the coloration of the molecules represents the log ratio of the gene fold changes. Green indicates reduced expression and red indicates increased expression

Since TGF-β centric networks appeared as the most common top network among the four drug conditions, we constructed a merged TGF-β network containing the combination of all genes present in TGF-β centric networks (Figs. 4 and 5). This network merged the top networks from all four treatments that contain a member of the TGF-β family. The three TGF-β elements of this network are TGFB1, TGFB3, and the more general TGF-β family. PNF-1 treatment results in the upregulation of all three TGF-β elements, as well as numerous downstream targets (Fig. 4a). In addition, basic fibroblast growth factor (bFGF) and CLCA2 were highly upregulated. In contrast, SC-3–141 treatment that had almost twice the effect on HUVEC proliferation results in activation of just the TGF-β family, as well as COL1A1, EIF3, and PDGF BB. It also results in downregulation of FGF (Fig. 4b). SC-3–143 treatment upregulates the TGF-β family as well as TGFB3 elements (Fig. 5a). P13K, IFIT1, SERPINF2, and KCNIP2 were also upregulated following SC-3–143 treatment. SC-3­263 treatment results in the upregulation of KRT14, TGFB1, and TGF-β family (Fig. 5b). It also downregulated PDGF BB and upregulated FGF. Despite the slight differences in network activation, this merged network demonstrates the homogeneity of the TGF-β pathways in the proposed mechanism of these pthalidomide analogues.

### Compendium Analysis Application Confirms Interactions with TNF-α and Suggests Interplay of NR3C1, PDGF, and bFGF

**Fig. 6:**
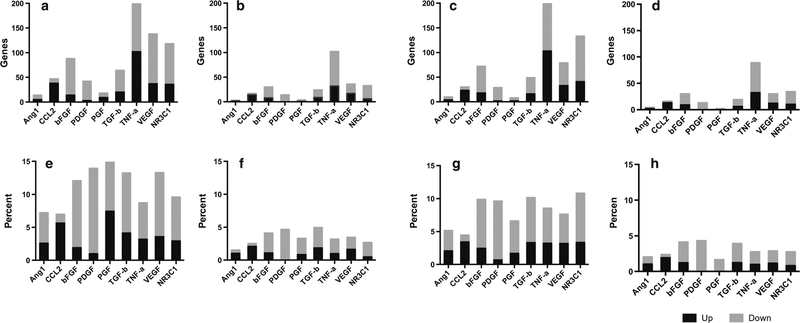
24-h compendium analysis. Compendium analysis with the addition of NC3C1 (glucocorticoid receptor signaling) is applied to the 24-h time points of 4 drugs (**a** SC-3–141, **b** SC-3–143, **c** SC-3–263, **d** PNF-1). **a–d** The application of the compendium to all four groups, with results shown as total genes. All groups show elevated numbers of TNF-a, pathway activation, while SC-3–141 and SC-3–263 both show significant NR3C1 and VEGF pathway activity. **e–h** The application of the compendium with results shown as percentage of pathway activation. The TNF-α pathway appears much less pronounced using this normalized method. All pathways with the exception of CCL2 shown higher levels of downstream activation than upstream; however, bFGF and PDGF show a much larger percentage difference between up- and downstream activation

The network compendium applied to all four datasets at the 24-h time point shows that the TNF-α pathway had the highest total expression across all groups, and the NR3C1 pathway was second highest for all groups except SC-3–141, which demonstrated elevated VEGF activity. All pathways except CCL2 had higher levels of downstream activation than upstream activation (Fig. 6). Additionally, overall gene expression for SC-3–141 and SC-3–263 was higher for all compendium pathways than either PNF-1 or SC-3–143.

The compendium was then applied at all seven time points to PNF-1 (Fig. 9). This allowed for the analysis of the temporal profile of NR3C1 with the addition of the new molecular interactions added to IPKB since 2008. Results show elevated TNF-α pathway activation, especially in the 2-, 4-, and 48-h time points. This corresponds with the previous findings that TNF-α is instrumental to vessel formation by PNF-1. TGF-β activation did not appear as prominent as previously found, although network analysis still identified TGF-β as crucial to the mechanism of action for PNF-1. NR3C1 displayed the second highest total activation in six out of the seven time points, further suggesting its importance in the mechanisms of pthalidomide-based molecules.

### PLSR Model with Variables Obtained from Compendium Analysis Predicts Cell Proliferation

**Fig. 7:**
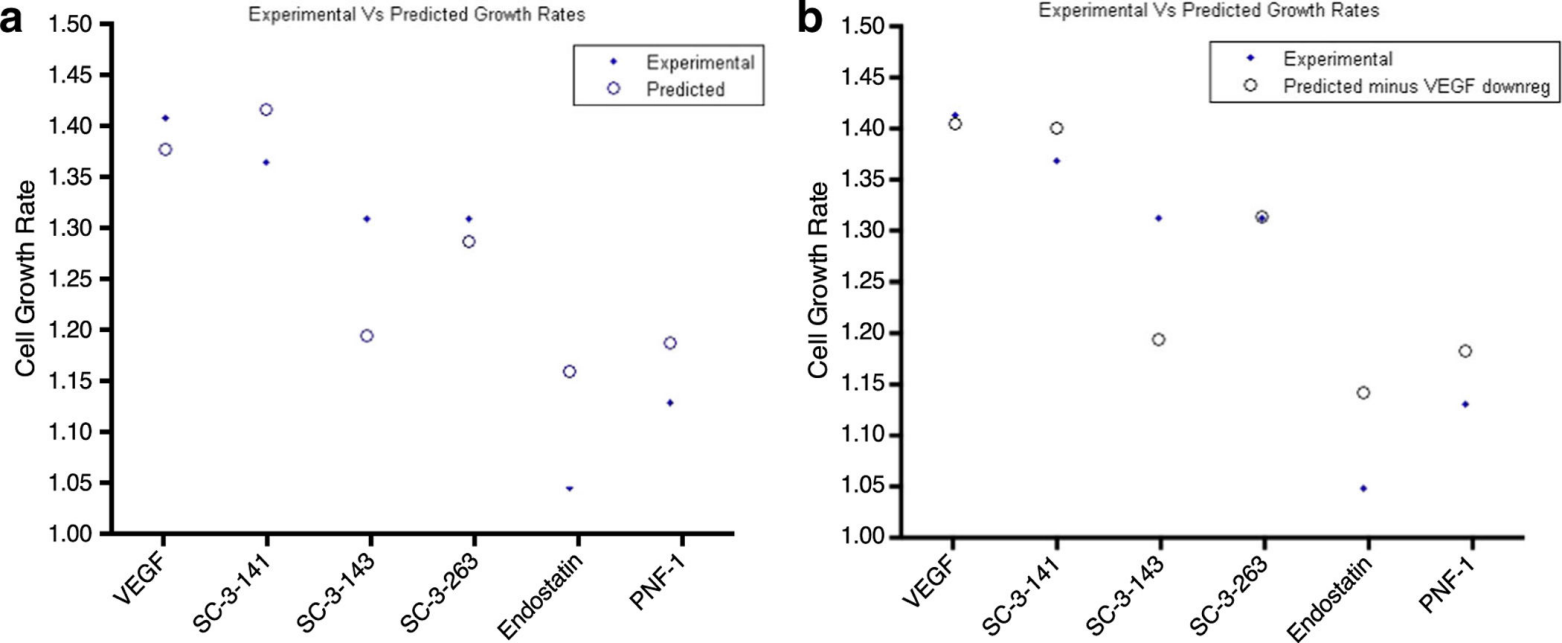
PLSR modeling. Figure 6: PLSR modeling. **a** The initial output from using the gene data from the first five variables to predict all the cell growth data for all six molecules. **b** The prediction results post-removal of prediction data that was identified in sensitivity testing. Removing the VEGF data points resulted in a better prediction for PNF-1, the only drug not included in the prediction data. It also improved the prediction of the other drugs

Initially, all 18 variables from the compendium analysis from 5 drugs (SC-3–141, SC-3–143, SC-3–263, VEGF, and endostatin) were used to predict cell proliferation after 24 h of stimulation for all 6 drugs (SC-3–141, SC-3–143, SC-3–263, VEGF, endostatin and PNF-1) (Fig. 7a). We analyzed this model by performing a sensitivity analysis that showed that VEGF accounts for a high percentage of error in the model. Therefore, after applying the same model with the removal of VEGF as an input variable, the error percent decreases (Fig. 7b). This resonates with our previous conclusion that these molecules are pro-angiogenic in a VEGF-independent manner and that the genes activated up- or downstream, as identified by the compendium analysis, can be used to predict cellular behavior. However, we should note that the percent error of this predictive model for endostatin and SC-3–143 are unusually high which could indicate intrinsic differences in the gene network of these two drugs compared to the others.

## Discussion

Structural adaptation and stability of microvascular networks is critical to many therapeutic applications, particularly those in which immediate nutritive and metabolic demands are generated [6, 16, 17]. Microvascular remodeling is a complex emergent phenomenon arising from the aggregate of individual cellular fates, such as migration, proliferation, differentiation, and apoptosis that are ultimately controlled by genome expression patterns and cues from the local microenvironment. Strategies for induction of neovascularization are often inferred from the effects of over-expressing or under­expressing individual members of known angiogenic signaling pathways, many times without addressing potential compensatory or cooperative effects between signals and the over­arching signaling networks that regulate changes in cell function. The challenge remains to harness the expanding knowledge base of molecular regulators of angiogenesis for development of successful pro-angiogenic therapies.

PNF-1 and our three-novel pthalidomide analogues are distinctive drugs under development for therapeutic angiogenesis and regenerative medicine. Uncovering the mechanisms of action of these potentially therapeutic drugs is critical for commercialization and safe integration into clinical settings. PNF-1 has previously been investigated using in vivo angiogenic assays and network analysis tools [9–11]. These investigations suggested that PNF-1 induced angiogenesis through the use of the pro-inflammatory cytokine TN F-α and the growth factor TGF-β. In this study, we investigated PNF-1 alongside three-novel pthalidomide analogues due to their ability to promote angiogenesis despite the severe downregulation of VEGF pathways. Pthalidomide analogues traditionally inhibit angio-genesis due to the complete inhibition of VEGF pathways, among other factors [18]. This has led to their development as anti-cancer drugs that inhibit tumor vascularization. Pthalidomide analogues also traditionally reduce TNF-α and other pro-inflammatory cytokines such as IL-2, – 8, and – 12 [19]. Our use of gene network and functional analysis shows that the reason for many of these inconsistencies between the pthalidomide analogues tested here and others is because of the activation of TGF-β and NR3C1 pathways.

TGF-β pathways were identified to be a critical component in all four pthalidomide analogue mechanisms. TGF-β is known to induce angiogenesis through PDGF BB and bFGF [20, 21]. This is consistent with our findings in the merged network, since IPA identified both PDGF and bFGF to be part of the network, and both were activated across multiple conditions (Fig. 2). Also, both PDGF and bFGF were identified by the compendium analysis in Fig. 6 as having high percentages of downstream activity, suggesting that these pathways were somehow activated independent of their usual upstream regulators. Additionally, the homogeneity of NR3C1 across conditions suggests that it also plays a significant role in the cellular response. Glucocorticoids have been shown to induce bFGF and other growth factors in neurons [22]. The upregulation of NR3C1 in HMVECs following treatment indicates that the pthalidomide analogues may activate glucocorticoid pathways, resulting in the bFGF response. This is consistent with the findings from the compendium results in Fig. 6, suggesting that the downstream activation in the bFGF pathways could be the result of NR3C1 upregulation. Additionally, we have also shown that the genes identified from compendium analysis can be used to predict the proliferation rates of HMVECs after 24-h treatment. This indicates that a compendium pathway analysis can be paired with a PLSR model to accurately predict cellular behavior.

**Fig. 8:**
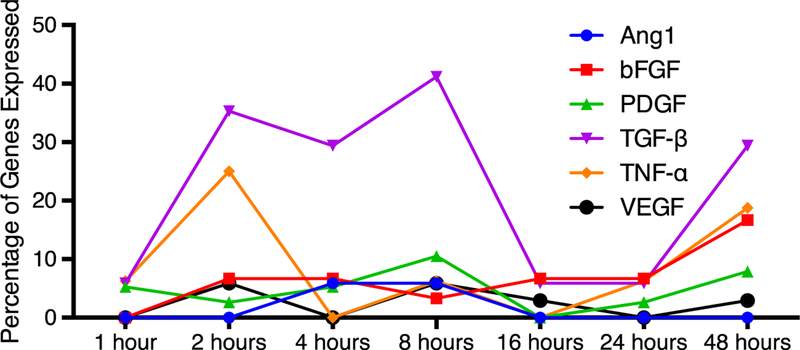
NCI PID compendium comparison for PNF-1 treatment. The alternative pre-processing method shows an overall decrease in differential expression across most time points compared to that presented previously. However, there is a notable increase in TGF-β at the 2-h time point. The general trend remains the same however, with expression spikes at 2, 8, and 48 h

The re-application of the pathway compendium to the temporal profile of PNF-1 reaffirms the importance of TNF-α signaling in the physiological responses. Previous research implicates TN F-α induction of TGF-β through the ERK-specific MAPK pathway and AP-1 activation [23–25]. The NR3C1 pathway appears as the second highest activated pathway after TNF-α, which once again suggests the integration of NR3C1 into the established TNF-α and TGF-β mechanisms. Finally, spikes in gene expression at the 2-, 8-, and 48-h time points reveal a pattern of gene expression that may allow for more specific temporal targeting in future experiments (Fig. 8). Pairing gene expression data with simultaneous in vitro or in vivo physiological data could elucidate whether this change in gene expression corresponds to cellular or physiological changes.

**Fig. 9:**
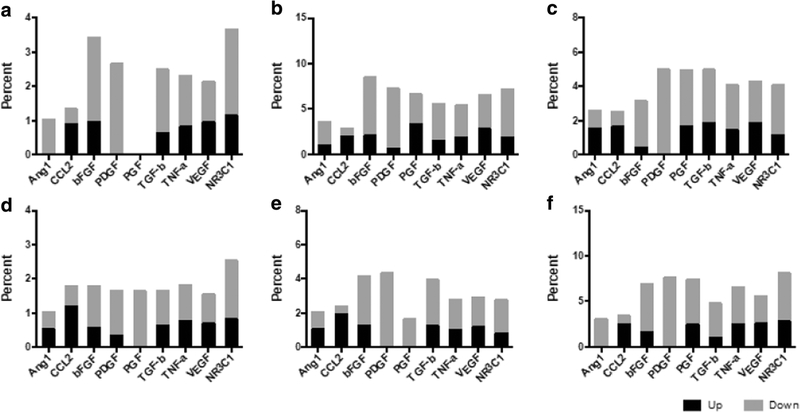
PNF-1 temporal compendium. The application of the compendium analysis with the addition of NC3C1 (glucocorticoid receptor signaling) to all seven time points of PNF-1 treatment. **a–f** The application of the compendium with results shown as percentage of pathway activation. TNF-α pathway appears much less pronounced using this normalized method. All pathways except for CCL2 show higher downstream than upstream activation, although PDGF by far shows the highest ratio of up-to-downstream regulation; (**a** hour 1, **b** hour 2, **c** hour 8, **d** hour 16, **e** hour 24, **f** hour 48)

In conclusion, the pairing of compendium pathway analysis with the IPKB analysis is a comprehensive technique that aims to reduce bias in approaching suggestions for drug mechanisms. Looking for similarities between techniques and confirming interactions via literature served to link the two techniques and provide a more comprehensive analysis. This could serve as a useful framework to harness the vast knowledge base of angiogenic signaling pathways to develop successful new therapies. Both techniques proved useful in identifying the key gene targets of the pthalidomide analogues: TGF-β, TNF-α, and NR3C1. Though the likelihood of identifying a unifying common pathway for all four analogues was not likely, enough similarities exist for a number of generalizations to be made. The homogeneity of TGF-β and NR3C1 activation between groups mixed with the downstream activation of PDGF and bFGF activation suggests a complex mechanism that acts independent of the common VEGF pathway (Fig 9).

## Figures and Tables

**Table 1:** Differential and focus genes

	2008	2013			
	PNF1	PNF1	SC-3-141	SC-3-143	SC-3-263
Differentially expressed genes	568	628	1377	437	1273
Focus genes	264	508	1057	320	1022
Percent	46.50	80.90	76.80	73.20	80.30

Differentially expressed genes (from HMVECs treated with 30-μM drug) after pre-processing and selection by IPA based on eligibility for network analysis. Comparison between old and new analysis for PNF-1 shows a much higher percentage of gene eligibility in IPA

**Table 2:** Common nodes

	PNF-1	SC-3-141	SC-3-143	SC-3-263
PNF-1	*539*	52	8	50
SC-3-141	52	*1211*	52	215
SC-3-143	8	52	*331*	64
SC-3-263	50	215	64	*1105*

Comparison of the shared nodes (focus genes) between each drug treatment group. Italicized numbers are the total focus gene number for each treatment, while non-italicized numbers show the commonalities between compounds

**Table 3:** Activated upstream regulators

	PNF-1	SC-3-141	SC-3-143	SC-3-263
PNF-1	*16*	3	1	0
SC-3-141	3	*86*	9	7
SC-3-143	1	*9*	*118*	35
SC-3-263	0	*1*	35	*105*

Upstream regulator molecules and proteins that are predicted to be activated based on the expression levels of their downstream targets. Activation is inferred by Ingenuity based on *z*−scores that takes in to account overlapping *p* values from known gene targets in each dataset. Italicized numbers show the activated totals for each compound, while non-italicized numbers show the commonalities between compounds

**Table 4:** Inactivated upstream regulators

	PNF-1	SC-3-141	SC-3-143	SC-3-263
PNF-1	*10*	1	0	0
SC-3-141	1	*63*	2	1
SC-3-143	0	*2*	*17*	4
SC-3-263	0	1	4	*26*

Upstream regulator molecules and proteins that are predicted to be inactivated based on the expression levels of their downstream targets. Inactivation is inferred by Ingenuity based on *z*−scores that takes in to account overlapping *p* values from known gene targets in each dataset. Italicized numbers show the activated totals for each compound, while non-italicized numbers show the commonalities between compounds

**Table 5:** Quantification of node connections in top networks

Drug	Network	Gene	Connections	Regulation
PNF-1	1	SPTAN1	6	Down
		EZR	10	Up
		P13K	15	–
		Alpha catenin	8	–
		Alpha actenin	9	Up
	2	TGFB1	25	Up
		INHBA	9	Down
		TMP2	5	Up
	3	RNA polymerase II	9	Up
		Histone h3	13	–
		SOD2	8	Down
SC-3-141	1	VEGF	18	Down
		MAD2L1	10	Down
	2	MYCN	9	Up
		NPM1	8	Down
		ACTB	8	Down
	3	SERPINE 1	11	Up
		NR3C1	19	Up
SC-3-143	1	IgG	19	Up
		NF-kB (complex)	18	–
		TNFSF13B	8	Up
	2	pl3k (complex)	16	Up
		TGF beta	14	Up
		TGFB3	9	Up
	3	BMP-4	12	Up
		ERK1/2	18	–
		SMAD1	10	Up
SC-3-263	1	FOS	26	Up
		SOS	20	–
	2	TUN	14	Up
		HNRNPA2B1	10	Down
		SnRNP	6	Up/down
		NNRNPA1	12	Down
	3	TGFB1	29	Up

Top scoring genes are listed with their corresponding number of molecular interactions as well as their activation state. These include intra-network connections as all inter-network connections are ignored
